# NMR Characterizations of the Ice Binding Surface of an Antifreeze Protein

**DOI:** 10.1371/journal.pone.0015682

**Published:** 2010-12-28

**Authors:** Jiang Hong, Yunfei Hu, Congmin Li, Zongchao Jia, Bin Xia, Changwen Jin

**Affiliations:** 1 Beijing NMR Center, Peking University, Beijing, China; 2 College of Life Sciences, Peking University, Beijing, China; 3 College of Chemistry and Molecular Engineering, Peking University, Beijing, China; 4 Department of Biochemistry, Queen's University, Kingston, Ontario, Canada; University of South Florida, United States of America

## Abstract

Antifreeze protein (AFP) has a unique function of reducing solution freezing temperature to protect organisms from ice damage. However, its functional mechanism is not well understood. An intriguing question concerning AFP function is how the high selectivity for ice ligand is achieved in the presence of free water of much higher concentration which likely imposes a large kinetic barrier for protein-ice recognition. In this study, we explore this question by investigating the property of the ice binding surface of an antifreeze protein using NMR spectroscopy. An investigation of the temperature gradient of amide proton chemical shift and its correlation with chemical shift deviation from random coil was performed for CfAFP-501, a hyperactive insect AFP. A good correlation between the two parameters was observed for one of the two Thr rows on the ice binding surface. A significant temperature-dependent protein-solvent interaction is found to be the most probable origin for this correlation, which is consistent with a scenario of hydrophobic hydration on the ice binding surface. In accordance with this finding, rotational correlation time analyses combined with relaxation dispersion measurements reveals a weak dimer formation through ice binding surface at room temperature and a population shift of dimer to monomer at low temperature, suggesting hydrophobic effect involved in dimer formation and hence hydrophobic hydration on the ice binding surface of the protein. Our finding of hydrophobic hydration on the ice binding surface provides a test for existing simulation studies. The occurrence of hydrophobic hydration on the ice binding surface is likely unnecessary for enhancing protein-ice binding affinity which is achieved by a tight H-bonding network. Subsequently, we speculate that the hydrophobic hydration occurring on the ice binding surface plays a role in facilitating protein-ice recognition by lowering the kinetic barrier as suggested by some simulation studies.

## Introduction

Antifreeze protein (AFP), discovered decades ago [1], is widely exploited by a variety of organisms to deal with the harsh condition at freezing temperatures [2], [3]. Antifreeze proteins are generally thought to work by an absorption-inhibition mechanism [4], [5], with bound protein causing interfacial curvature that leads to a reduction in local freezing temperature due to Kelvin effect [5]. For moderately active AFP, such as type I fish AFP, sufficient ice binding affinity apparently can be contributed by either H-bonding or hydrophobic interactions as suggested by various mutagenesis studies [6]–[8]. For hyperactive AFP such as *Tenebrio molitor* AFP (TmAFP) and spruce budworm AFP (sbwAFP), a strong binding affinity is gained dominantly from the near perfect lattice match of the ice oxygen atoms with the H-bonding groups on a two-dimensional array of threonine (Thr) residues on a flat β-sheet region [9], [10]. A remaining question is how protein specifically recognizes ice surface over bulk water which is at much higher concentration than ice nuclei. It is hard to tackle this question experimentally, whereas various simulation studies pointed out the occurrence of hydrophobic hydration on ice-binding surface, and/or suggested that the interfacial water is mediating protein-ice recognition [2], [11]–[16]. In this study, we explore this question by investigating the structural/dynamic property of the ice-binding surface of an antifreeze protein using NMR spectroscopy.

In this study, the hyperactive insect antifreeze protein, CfAFP−501, was chosen, for which the solution and crystal structures are available [17], [18]. Like other insect antifreeze proteins such as TmAFP and sbwAFP, CfAFP−501 forms a highly regular β-helix structure. The repeating sequence Thr-Xaa-Thr, which was confirmed to be the ice binding surface [9], can make near perfect lattice match with the oxygen atoms on ice. The overall rigidity of insect AFP, resulting from the highly regular β-helical structure with a large number of disulfide bonds and an extensive H-bond network, may facilitate a good lattice match in protein-ice complex formation. In this study, we characterized the structural/dynamic property of the ice binding surface by analyzing the temperature gradient of amide proton chemical shift and its correlation with chemical shift deviation from random coil reference, in combination with hydrogen-deuterium (H-D) exchange and relaxation measurements. Our results suggest the existence of certain amount of hydrophobic hydration on the ice binding surface of the protein.

## Results

**Figure 1:**
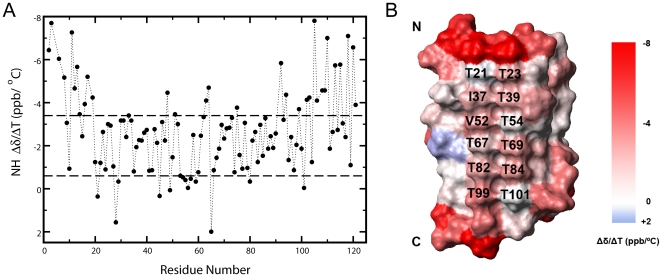
Temperature gradient of chemical shift (Δδ/ΔT) of CfAFP-501 amide protons. (A) Plotted versus residue number, the amide proton Δδ/ΔT (ppb/°C) over temperature range 0–30°C mostly falls into the range −2.0±1.4 ppb/°C. (B) Mapping of Δδ/ΔT onto the protein surface. The two rows of Thr residues in the Thr-Xaa-Thr motif are labeled, with the left row designated as the first Thr row and the right one as the second Thr row.

The NMR chemical shift is sensitive to the chemical environment of the observing nuclei and its temperature gradient contains information on temperature associated structural or dynamic changes [19]. To characterize the structural/dynamic property of CfAFP−501, especially its potential change with temperature, a combined analysis of the temperature gradient of amide proton chemical shift (Δδ/ΔT) with chemical shift deviation (CSD) [20] was performed. Figure 1 shows the Δδ/ΔT values as a function of residue number, and maps the values onto the protein surface. Clearly, most residues fall in the range of −2.0±1.4 ppb/°C, which is typical for H-bonded exchange-protected amide protons according to statistical analysis of protein and peptides [20]. This indicates that most amide protons of the protein are H-bonded over the temperature range investigated (0–30°C) except those at the N- and C- termini [20]. This result is in agreement with H-bond calculation from crystal and solution structures [17], [18].

**Figure 2:**
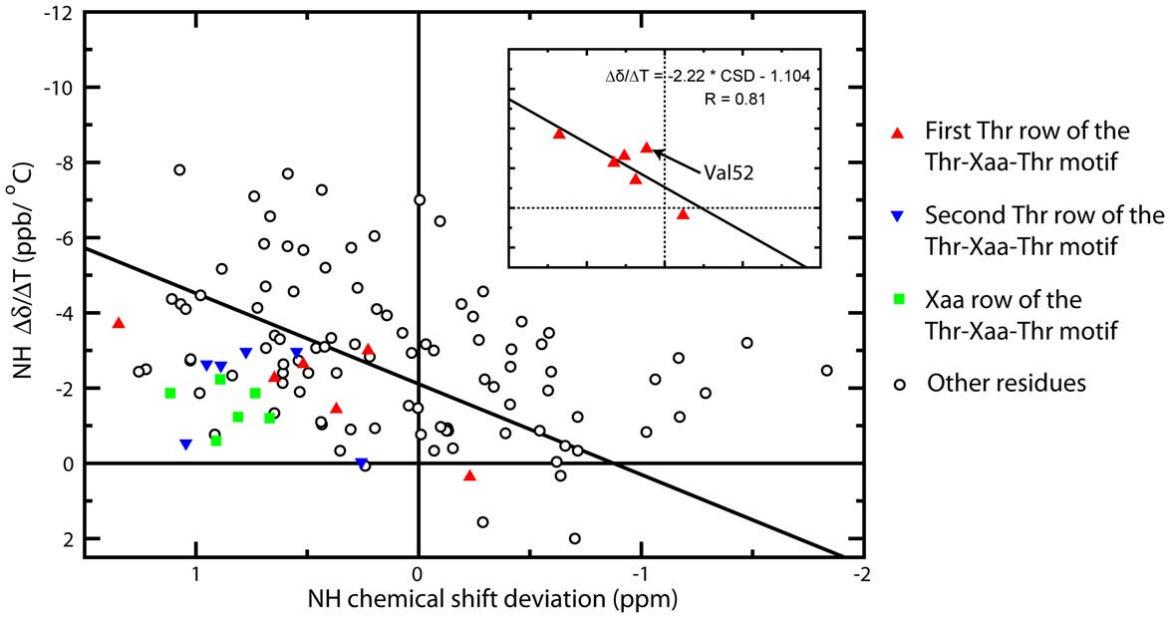
The amide proton Δδ/ΔT-CSD correlation plot of CfAFP-501. The statistical cutoff line, Δδ/ΔT  = −2.41× CSD −2.11, is drawn. The inset shows the amide proton Δδ/ΔT-CSD correlation plot for the residues of the first Thr row.

The correlation plot of Δδ/ΔT versus CSD can be used to determine the slow-exchanging H-bonded sites with much higher confidence than the Δδ/ΔT value alone [20]. The correlation plot is presented in Figure 2, for which the same format is taken as in [20] with downfield amide proton resonances placed to the left and amides displaying larger upfield shifts upon warming placed toward the top of the graph. As shown in Figure 2, the residues in the Thr-Xaa-Thr motif on the ice binding surface mostly fall into the lower left-hand corner of the correlation plot and are all below the statistical cutoff line: Δδ/ΔT  = −2.41× CSD −2.11. Taken together, the two features suggest that these residues are H-bonded and slow-exchanging based on previous analysis [20]. The observation that the Thr-Xaa-Thr motif comprises mostly of the lower left-hand corner of the plot (hence significantly deviates from random coil value) may imply that the ice binding surface has less motional flexibility than other parts of the protein surfaces. Intriguingly, the first Thr row exhibits a good correlation between Δδ/ΔT and CSD with correlation coefficient R value of 0.81 (0.96 if one apparent outlier, Val52, is excluded), as shown in the inset of Figure 2. Unlike the first Thr row, the second Thr row or the Xaa row in the Thr-Xaa-Thr motif does not show significant correlation between Δδ/ΔT and CSD values. Moreover, residues on the other two rectangular surfaces (recall that the β-helical structure of CfAFP-501 has a triangular cross-section) are completely uncorrelated for their Δδ/ΔT and CSD values (R value of 0.1–0.3).

**Figure 3:**
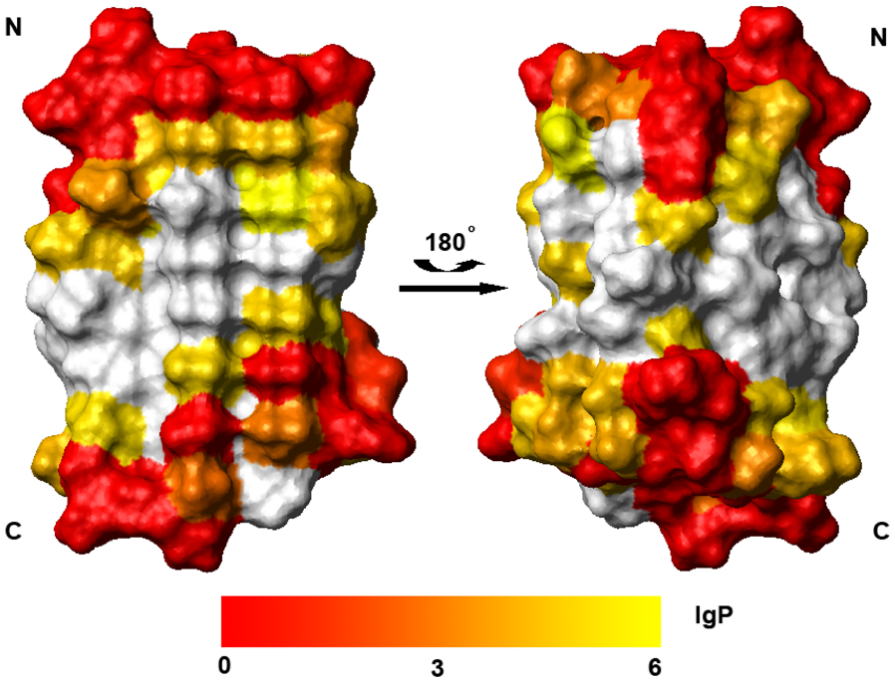
Mapping of the amide proton protection factor at 17°C onto the surface of CfAFP-501. Residues with low protection factor are colored red, and residues with high protection factor in white. The left view has the orientation showing the ice-binding surface.

We carried out hydrogen-deuterium exchange experiments to investigate possible local conformational fluctuation for CfAFP−501 at non-denaturing conditions. Our data at 5, 17, and 30°C showed a similar pattern of amide proton protection. The protection factors measured at 17°C are mapped onto the protein surface as shown in Figure 3. Most residues except those at the N- and C-termini show large values of protection factor (P>10^3^), indicating inaccessibility to solvent. Moreover, all residues except Thr84 and Thr99 in the Thr-Xaa-Thr motif have P values in the range of 10^3^–10^6^. Such high protection factors indicate that no significant local conformational fluctuation exists in most parts of the protein including ice binding surface. These protected protons are most likely involved in H-bonding due to the regular β−helical structure of insect AFPs.

**Figure 4:**
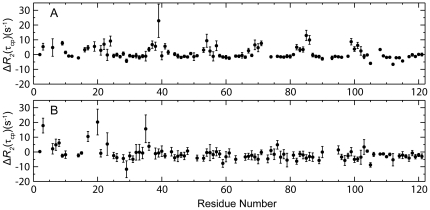
Conformational exchange of CfAFP-501 observed at 17°C (A) but not at −3°C (B). The difference of apparent R_2_ at τ_cp_ value of 1.0 and 10.0 ms, i.e., ΔR_2_(τ_cp_)  =  R_2_(10.0 ms)- R_2_(1.0 ms), is plotted versus residue number.

A weak dimer formation of CfAFP-501 at room temperature was suggested by rotational correlation time analysis and further supported by relaxation dispersion measurements. We measured the backbone ^15^N longitudinal and transverse relaxation rates R_1_ and R_2_ of CfAFP-501 and calculated the rotational correlation time τ_c_ by rotational diffusion tensor analysis. Only fairly rigid residues that display neither ps-ns timescale flexibility nor µs-ms timescale conformational exchanges were chosen for the diffusion tensor calculation. The results showed that the τ_c_ values of CfAFP-501 are 10.3±0.5 ns and 15.8±1.8 ns at 17 and −3°C, respectively. An estimation of the τ_c_ values of the protein monomer, using the program HYDRONMR [21] and the monomeric structure of CfAFP-501 (PDB 1Z2F) and considering the temperature-associated solution viscosity changes, gives τ_c_ values of 8.6 ns and 16.9 ns at 17 and −3°C, respectively. The ratio of τ_c_ (17°C)/τ_c_ (−3°C) is about 2 for monomeric CfAFP-501 based on HYDRONMR calculation, comparing to 1.5±0.2 determined from the relaxation data. Our data hence suggest weak dimer formation at 17°C and an equilibrium shift to monomer at lower temperature. The notion of dimer formation is further supported by relaxation dispersion measurement using TROSY-CPMG pulse program [22]. We measured the apparent R_2_ of CfAFP-501 at three τ_cp_ values, 1.0, 5.0, and 10.0 ms, and calculated the difference ΔR_2_(τ_cp_) ( = R_2_(10.0 ms)- R_2_(1.0 ms)) at both 17 and −3°C (Figure 4). The ΔR_2_(τ_cp_) values at 17°C but not at −3°C show a clear periodicity versus the protein sequence. The residues showing positive values of ΔR_2_(τ_cp_) are located in or close to the Thr-Xaa-Thr motif, indicating that the ice binding surface is involved in the conformational exchanges. The distinct results observed at 17°C and −3°C support the scenario of a dimer-to-monomer transition at lower temperature.

## Discussion

### Temperature-dependent protein-solvent interaction on ice binding surface

In this study, a good correlation between the temperature gradient of amide proton chemical shift and CSD is observed for the first Thr row on the ice binding surface. A good correlation between Δδ/ΔT and CSD is generally an indication of concerted conformational transition over temperature range investigated [20]. However, explanations other than structural transition are plausible for interpreting the observed correlation here, on the grounds of the following. First, residues in the first Thr row are H-bonded over the temperature range investigated. This leaves minimal possibility for cooperative conformational transition involving amide protons. Second, it cannot be explained by dimer-monomer transition since otherwise it would require more dimer formation at low temperature instead of at high temperature as observed here. Third, the slope for this correlation, −2.22, is considerably smaller than those observed for cooperative conformational transition in peptides, but fall into the range normally observed for residual correlation in stable globular proteins (−1.4 - −4.1 ppb/°C) [20]. For the latter case, possible contributing factors include temperature-dependent protein-solvent interaction, change of population distribution of side chain rotamer, and change of backbone vibration with temperature [20]. Based on the following reasons, we suggest that the temperature-dependent protein-solvent interaction is most likely the dominant contributing factor to the observed correlation. First, for a structurally similar β-helical antifreeze protein TmAFP, it was found that all Thr side chains have exactly the same rotameric conformation [10] and the motions of these side chains are highly restricted [23]. The situation is the same for CfAFP-501 [18]. Second, the contribution to the observed correlation from either backbone vibration or side chain rotamer population change cannot be dominant otherwise it would be hard to reconcile the finding that the second Thr row does not have such correlation. Similarly, the lack of correlation on the other two rectangular sides (non-ice binding surfaces) or on the Xaa row in the Thr-Xaa-Thr motif and possession of a correlation on the first Thr row can hardly be explained by different nature of backbone vibration.

Significant temperature-dependent protein-solvent interaction for ice-binding surface points to a scenario of hydrophobic hydration. By analogy with the correlation resulting from temperature-dependent conformational exchange, the observed correlation here suggests that corresponding hydration water is more structured at low temperature and hence implies stronger protein-solvent interaction (and hence stronger hydration) at low temperature. A stronger hydration at lower temperature is consistent with a scenario of hydrophobic hydration, for which the typically large positive hydration heat capacity change can be explained by postulating a rapid hydration decrease as temperature increases. The different behavior between the two Thr rows may reflect subtle difference in local surface property and associated hydration water. The second Thr row is strictly conserved while the first row can accommodate residues with nonpolar side chains [24]. A subtle different dynamics behavior between the two Thr rows was also reported for TmAFP [25].

### The hydrophobic nature of CfAFP−501 ice binding surface

In this study, CfAFP−501 is observed to form a weak dimer through its ice binding surface and experiences a dimer to monomer shift as temperature decreases. The following qualitative thermodynamic analysis of this behavior suggests hydrophobic hydration involved in ice-binding surface. Considering the monotonous part of dimer affinity curve as a function of temperature, i.e., the part before the affinity reaches its maximum, we can perform a qualitative thermodynamic analysis to derive the nature of the dimer formation process. More dimer formed at high temperature than at low temperature implies a negative slope of dimer-formation free energy with respect to temperature, which indicates a positive entropy change for dimer formation. Meanwhile, an approximate Van't Hoff analysis on the temperature effect on dimer affinity reveals an enthalpically unfavorable dimerization. Therefore, the dimer formation is driven by positive entropy change. This favorable entropy change associated with dimerization must result dominantly from hydrophobic effect, like most protein-ligand interactions, since the contribution from dehydration of polar groups are negligible [26]. The dimer interface, found to be the same as the ice binding surface, therefore shows certain hydrophobic feature and involves hydrophobic hydration at monomeric state. A weak dimer formation and a similar temperature-dependent dimer-monomer equilibrium shift were also reported for sbwAFP [27]. The nature has obviously avoided too much hydrophobicity for the ice binding surface otherwise dimerization still exists at low temperature which inhibits AFP function. Probably that is why the nature has chosen Thr instead of Ser for ice binding surface.

In summary, we observed a good correlation between the temperature gradient of amide proton chemical shift and CSD for one of the Thr rows on ice binding surface, and observed a weak dimer formation through ice binding surface at room temperature and dimer-to-monomer transition at low temperature. Both observations suggest hydrophobic hydration involved in ice binding surface. Our finding is consistent with simulation studies for various antifreeze proteins where hydrophobic hydration was found on ice binding surface but not on non-ice binding surface [11], [13], [14], [16]. Hydrophobic hydration of ice-binding surface may also occur on another two insect AFPs, TmAFP, for which a similar correlation between Temperature gradient of amide proton chemical shift and CSD can be seen from the reported data [25], and sbwAFP, for which a weak dimer formation at high temperature and dimer-to-monomer transition at low temperature was also reported [27]. This hydrophobic hydration found on the ice binding surface is obviously not evolved for enhancing ice binding affinity. As pointed out [9], [10], a strong binding affinity is obtained from the near perfect lattice match of the ice oxygen atoms with the H-bonding groups on a two-dimensional array of Thr residues on a flat β-sheet region. We speculate that the hydrophobic hydration of the ice binding surface is involved in initial protein-ice recognition by lowering the kinetic barrier as also suggested by recent simulation studies [13], [14].

## Materials and Methods

### Protein sample preparation

The expression, refolding, and purification procedure of CfAFP−501 is similar to the protocol published previously [18], [28], [29]. Briefly, uniformly ^15^N labeled protein was expressed in *Escherichia coli* BL21(DE3)/pLysS cells grown in M9 minima media using ^15^N labeled ammonium chloride as the sole nitrogen source. Protein samples for NMR measurements were prepared in 50 mM phosphate buffer (pH 5.7, with 90% H_2_O/10%D_2_O and 50 mM NaCl), and 2,2-dimethyl-2-silapentane-5-sulfonic acid (DSS) was added as the internal reference. Protein concentration was about 0.2 mM. Previously published backbone assignments [28] were used in this study. Under the buffer condition used, the solution freezing point is −13°C in the presence of 0.2 mM CfAFP-501, which ensures a solution state of our working system at the lowest temperature studied (−3°C).

### Temperature gradient analysis of amide proton chemical shift and its correlation with chemical shift deviation from reference random coil

The 2D ^1^H−^15^N HSQC spectra of CfAPF-501 at different temperatures were acquired on a Bruker Avance 600 MHz spectrometer. Spectral widths of 8389.9 Hz for ^1^H and 1824.6 Hz for ^15^N were used, and 512 (^1^H) and 128 (^15^N) complex data points were collected with 64 transients per increment. At each temperature, the protein sample was allowed to equilibrate for 30 min. The spectra were processed using NMRPipe [30] and analyzed using NMRView [31]. The values of amide proton Δδ/ΔT were determined from the measured chemical shifts of backbone NH signals in the ^1^H−^15^N HSQC spectra over the temperature range 0–30°C with 5°C increment; linear variation of chemical shift with temperature was observed. The values of amide proton CSD ( =  δ_obs_ − δ_rc_) at 0°C were calculated from the observed chemical shifts (δ_obs_) and the corresponding reference values in random coil (δ_rc_), the latter taken from literature [20] with temperature correction to 0°C using a temperature coefficient for each type of amino acid [32] and with sequence position correction performed as described [20].

### Hydrogen-Deuterium exchange measurement

In this study, the H-D exchange experiments were carried out at 5, 17, and 30°C. Lyophilized ^15^N-labeled CfAFP-501 protein was dissolved in D_2_O (in 50 mM phosphate buffer, pD 5.7), and a series of 2D ^1^H−^15^N HSQC spectra were recorded as a function of time. The exchange rates (k_ex_) were determined by fitting the time-dependent peak intensity to a single exponential function.

The exchange rates were analyzed assuming an EX2 condition to derive information on proton accessibility or protection factor (P). The exchange reaction can be described by eq 1
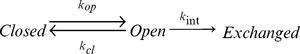
(1)where k_op_, k_cl_, and k_int_ are rate constants for opening, closing, and intrinsic exchange. For a stable protein structure under mild condition as in this study (no denaturant and not high temperature; no significant denaturation occurred as implied by HSQC spectra over temperature range investigated) (k_op_<<k_cl_), the exchange rate can be expressed as k_ex_ = K_op_k_int_ (K_op_ = k_op_/k_cl_) at EX2 limit (k_int_<<k_cl_) which is normally applicable for mild conditions [33]. Under this condition, a fast equilibrium is built between open and closed states with the equilibrium constant K_op_, which describes the extent of proton accessibility by the solvent and the inverse of which (namely the protection factor, P = 1/K_op_ = k_int_/k_ex_) reveals how well the proton is protected from the solvent. Protection factor P is therefore determined from the measured exchange rate k_ex_ and the intrinsic rate k_int_ calculated from unstructured peptides as described in [34], [35].

### Relaxation measurements

Backbone ^15^N relaxation parameters, including the longitudinal relaxation rate R_1_, transverse relaxation rate R_2_, and steady-state {^1^H}-^15^N heteronuclear NOEs [36] were measured using ^15^N-labeled CfAFP-501 sample on Bruker Avance 600 and 800 MHz spectrometers at temperatures of 17°C and −3°C, respectively. For the experiments acquired on the 600 MHz spectrometer, spectral widths of 8389.3 Hz for ^1^H and 1824.8 Hz for ^15^N were used. For the experiments acquired on the 800 MHz spectrometer, spectral widths of 11160.7 Hz for ^1^H and 2432.8 Hz for ^15^N were used. For the R_1_ and R_2_ measurements, 512 (^1^H) and 128 (^15^N) complex data points were collected with 32 transients per increment and a recycle delay of 2.5 s. The delays used for the R_1_ experiments were 11.52(x2), 81.52, 201.52, 351.52, 501.52, 651.52, 851.52, 1051.52, 1301.52, 1601.52, 2001.52, 2501.52 ms. The delays used for the R_2_ experiments were 6.14(x2), 14.42, 26.84, 35.12, 51.68, 64.10, 84.80, 101.36, 126.20 ms. The relaxation rate constants were obtained by fitting the peak intensities to a single exponential function and the uncertainties on the parameters were estimated by Monte-Carlo simulation. The {^1^H}-^15^N heteronuclear NOE experiments were performed in the presence and absence of a 3-s proton presaturation period prior to the ^15^N excitation pulse and using recycle delays of 5 and 8 s, respectively [36]. A total of 48 transients were used, and a duplicated set of experiment was recorded for experimental error estimation.

During the analysis of rotational diffusion tensor and estimation of the rotational correlation time, residues that exhibit either internal flexibility on ps-ns timescale or conformational exchanges on µs-ms timescale were excluded based on the following criteria. First, residues showing {^1^H}-^15^N heteronuclear NOE less than 0.75 have ps-ns timescale structural flexibility [37]. Second, the R_2_/R_1_ ratios for all residues were calculated, and those having R_2_/R_1_ ratios significantly larger than the average value (using 1.5 times standard deviation as a cut off) may have conformational exchanges on µs-ms timescale [37]. Finally, relaxation dispersion measurements by TROSY-CPMG were also used to identify residues exhibiting conformational exchanges on µs-ms timescale. Residues that fall into any of the above category were excluded from the analysis.

Relaxation dispersion measurements were performed to investigate possible conformational exchanges on µs-ms timescale using the TROSY-CPMG pulse sequence [22]. The experiments were performed on an 800 MHz Bruker Avance at 17°C and −3°C using 0.2 mM ^15^N-labeled protein. Spectral widths of 11160.7 Hz for ^1^H and 2432.8 Hz for ^15^N were used, and 512 (^1^H) and 128 (^15^N) complex data points were collected with 32 transients per increment and a recycle delay of 2.7 s. The τ_cp_ values of 1, 5, and 10 ms were used at both temperatures, where τ_cp_ is the delay between ^15^N π pulses in the CPMG pulse train.
